# Assessing the Risk of SARS-CoV-2 Transmission via Surgical Electrocautery Plume

**DOI:** 10.1001/jamasurg.2021.2591

**Published:** 2021-05-21

**Authors:** Leigh J. Sowerby, Anthony C. Nichols, Richard Gibson, Doron D. Sommer, Corey Moore, Douglas D. Fraser, Eric Arts

**Affiliations:** 1 Department of Otolaryngology–Head and Neck Surgery, University of Western Ontario, London, Ontario, Canada; 2 Department of Microbiology and Immunology, Western University, London, Ontario, Canada; 3 Department of Surgery, Division of Otolaryngology–Head and Neck Surgery, McMaster University, Hamilton, Ontario, Canada; 4 Department of Physiology and Pharmacology, Western University, London, Ontario, Canada; 5 Department of Otolaryngology–Head and Neck Surgery, Western University, St Joseph’s Hospital, London, Ontario, Canada

## Abstract

This quality improvement study used a nonhuman subject research approach to examine whether SARS-CoV-2 from aerosolized virus is present in and potentially transmissible from a electrocautery plume in surgery.

Live severe acute respiratory syndrome coronavirus 2 (SARS-CoV-2) virus has been detected in saliva, sputum, bile, feces, and blood and shown to remain viable in aerosols for at least 3 hours.^1,2^ As such, direct transmission to surgical staff from aerosolized virus in an electrocautery plume (as observed with other viruses) has been raised by several colleges and associations as a particular safety concern.^1,3^ Cautery performed in areas of high potential viral load in particular (eg, the nasopharynx, oropharynx, anterior skull base, lung parenchyma) could pose a risk to those in the operating room. Furthermore, sinonasal pathologies can mimic the symptom profile of COVID-19 and have been documented to contribute to false-negative nasopharyngeal screening results, further increasing potential perioperative risk and exposure.^4^

Respiratory RNA viruses with a lipid bilayer, such as SARS-CoV-2, are typically more susceptible to higher temperatures than other nonenveloped respiratory viruses, such as adenoviruses. Although SARS-CoV-2 loses infectivity at higher temperatures (eg, 70 °C) in media,^5^ inhalation of even small amounts of aerosolized virus appear sufficient to establish infection. However, tip temperatures of electrocautery range from 100 to 1200 °C, and as such, the temperature is potentially sufficient to inactivate SARS-CoV-2 in the plume.

## Methods

To examine this, we set out to investigate the presence of live SARS-CoV-2 in electrocautery plumes (eFigure in the Supplement) after an institutional review board waiver and approval was received from Lawson Health Research Institute. Electrocautery at 25 W was applied using 3 different methods (monopolar cut, monopolar coagulate, and bipolar electrocautery [Erbe USA]) for 1 minute on raw chicken breast with an added 4 mL of Dulbecco modified eagle medium (DMEM) or a DMEM:blood mixture containing 1 × 10^5.7^ median tissue culture infectious dose (TCID_50_) per mL of SARS-CoV-2, similar to the viral load in pulmonary sputum of a patient with symptoms. Each experimental condition was repeated in triplicate. An estimated volume of 1.7 ± 0.3 mL, 1.5 ± 0.1 mL, and 1.0 ± 0.2 mL of liquid was vaporized during the monopolar cut, monopolar coagulate, and bipolar electrocautery, respectively, and collected using a Western AirScan air sampler at 60 L per minute onto a gelatin filter in triplicate (Sartorius Canada). For a positive control, approximately 0.3 mL of both viral media and blood with SARS-CoV-2 was aerosolized (without heat) into the chamber and collected in the same fashion. The gelatin filters were solubilized in phosphate-buffered saline and added in undiluted and 1:10 serial dilutions to VeroE6 cells to determine the TCID_50_ value of the vaporized virus following electrocautery, as per the methods described by Bannerjee et al.^6^

## Results

Using a cell titer glow measurement for replicating virus,^6^ we observed no virus recovered from any electrocautery performed. However, collected aerosolized blood or media containing SARS-CoV-2 (approximately 0.3 mL) resulted in a recovery at least 3 or 4 base 10 logs higher than electrocautery or the negative control (Figure, A). The maximal theoretical recovery of SARS-CoV-2 on the gelatin filter was approximately 1 × 10^6.2^ units (or 1 × 10^9.2^ viral cytopathic effect units, from the cell titer glow measurement). Viral RNA was readily detected in the control aerosols of both fluids in the absence of cautery (Figure, B). The lack of SARS-CoV-2 was also confirmed by the lack of viral RNA on quantitative real-time polymerase chain reaction with undiluted vapor collected on the filter (Table).

**Figure.**
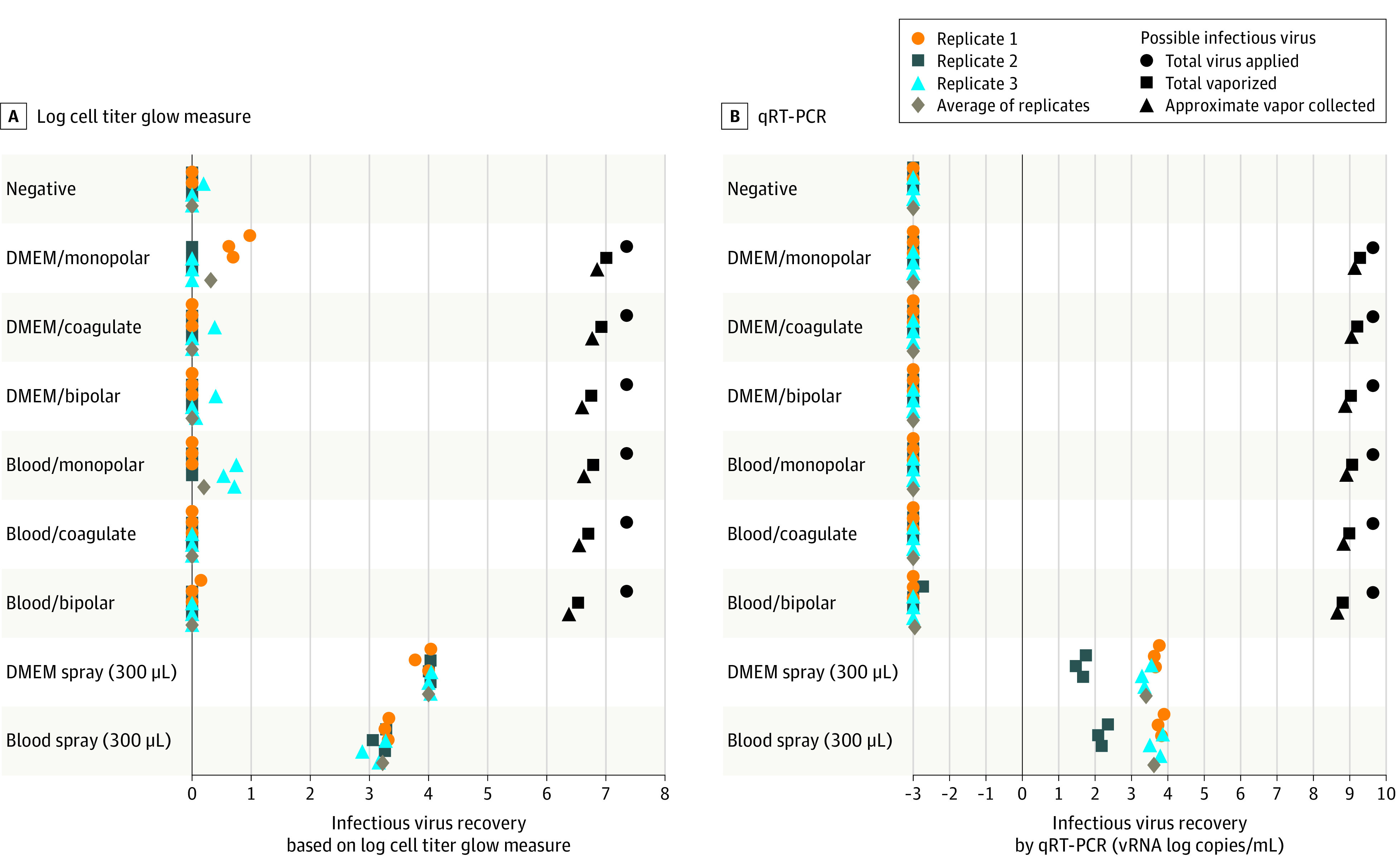
Cultured Severe Acute Respiratory Syndrome Coronavirus 2 Images A, Virus recovered, based on relative log10 relative cell titer glow measurements. B, Virus present, based on relative quantitative real-time polymerase chain reaction (qRT-PCR) measurements. DMEM indicates Dulbecco modified eagle medium; vRNA, viral RNA.

**Table.** Quantitative Real-Time Polymerase Chain Reaction (qRT-PCR) Analysis of COVID-19 Viral RNA on Filters^a^

Conditions	SARS-CoV-2 N gene RNA copies/mL												All replicates, mean (SD)	Viral cytopathic or infectious units/mL		
														Viral load applied to tissue	Theoretical viral load released with vapor^b^	Maximal theoretical viral amount
	qRT-PCR tests of biological replicate 1				qRT-PCR tests of biological replicate 2				qRT-PCR tests of biological replicate 3							
	1	2	3	Mean (SD)	1	2	3	Mean (SD)	1	2	3	Mean (SD)				
Negative control	<0.001	<0.001	<0.001	<0.001 (NA)	<0.001	<0.001	<0.001	<0.001 (NA)	<0.001	<0.001	<0.001	<0.001 (NA)	<0.001 (NA)	0	0	0
DMEM, monopolar cut	<0.001	<0.001	<0.001	<0.001 (NA)	<0.001	<0.001	<0.001	<0.001 (NA)	<0.001	<0.001	<0.001	<0.001 (NA)	<0.001 (NA)	2.16 × 10^10^	1.02 × 10^10^	7.11 × 10^9^
DMEM, monopolar coagulate	<0.001	<0.001	<0.001	<0.001 (NA)	<0.001	<0.001	<0.001	<0.001 (NA)	<0.001	<0.001	<0.001	<0.001 (NA)	<0.001 (NA)	2.16 × 10^10^	8.47 × 10^9^	5.93 × 10^9^
DMEM, bipolar electrocautery	<0.001	<0.001	<0.001	<0.001 (NA)	<0.001	<0.001	<0.001	<0.001 (NA)	<0.001	<0.001	<0.001	<0.001 (NA)	<0.001 (NA)	2.16 × 10^10^	5.64 × 10^9^	3.95 × 10^9^
Blood, monopolar cut	<0.001	<0.001	<0.001	<0.001 (NA)	<0.001	<0.001	<0.001	<0.001 (NA)	<0.001	<0.001	<0.001	<0.001 (NA)	<0.001 (NA)	2.16 × 10^10^	6.37 × 10^9^	4.46 × 10^9^
Blood, monopolar coagulate	<0.001	<0.001	<0.001	<0.001 (NA)	<0.001	<0.001	<0.001	<0.001 (NA)	<0.001	<0.001	<0.001	<0.001 (NA)	<0.001 (NA)	2.16 × 10^10^	6.77 × 10^9^	4.74 × 10^9^
Blood, bipolar electrocautery	<0.001	<0.001	<0.001	<0.001 (NA)	<0.001	<0.001	0.002	<0.002	<0.001	<0.001	<0.001	<0.001 (NA)	<0.001 (NA)	2.16 × 10^10^	5.42 × 10^9^	3.79 × 10^9^
DMEM control spray	5806	4225	4513	4848 (842)	56	46	29	44 (13)	3483	1920	2269	2557 (821)	2483 (2459)	NA	NA	NA
Blood control spray	7828	6598	5302	6576 (1263)	120	151	225	165 (54)	7118	6152	3173	5481 (2057)	4074 (3298)	NA	NA	NA

Abbreviations: DMEM, Dulbecco modified eagle medium; NA, not applicable.

^a^ It is assumed that the difference of amount added from the amount remaining would be the amount of liquid or virus released with the vapor plume.

^b^ The volumes of liquid added to the tissue were measured following cautery.

## Discussion

In this study, SARS-CoV-2 was not detectable in aerosol cautery plume generated from electrocautery under any of the conditions studied despite the high viral titers used. By mimicking surgery on a patient with a high SARS-CoV-2 load, there was a minimum of a 9 log reduction of viral RNA with any of the electrocautery methods. This suggests that electrocautery smoke is an unlikely source of SARS-CoV-2 transmission for health care workers. This study is limited by the in vitro nature of the experiment, and collecting cautery plumes from airway surgery in patients with active SARS-CoV-2 would be definitive. Future work investigating the plume associated with lower-temperature thermal surgery (such as coblation or carbon dioxide laser) and different tissue substrates is warranted.

## Notes

Supplement. **eFigure.** Schematic representation of COVID-19 electrocautery plume experiment
