# Supplementary material for: Assessing the Risk of SARS-CoV-2 Transmission via Surgical Electrocautery Plume
Source: JAMA Surg. 2021 May 21;156(9):883–5. doi: 10.1001/jamasurg.2021.2591 (PMC8140389; doi:10.1001/jamasurg.2021.2591)
Supplement: Supplement. — eFigure. Schematic representation of COVID-19 electrocautery plume experiment [file jamasurg-e212591-s001.pdf]

Supplemental Online Content

Sowerby LJ, Nichols AC, Gibson R, et al. Assessing the risk of SARS-CoV-2 transmission via surgical electrocautery plume. *JAMA Surg*. Published online May 21, 2021. doi:10.1001/jamasurg.2021.2591

eFigure. Schematic representation of COVID-19 electrocautery plume experiment

This supplemental material has been provided by the authors to give readers additional information about their work.

© 2021 Sowerby LJ et al. *JAMA Surgery*.

eFigure. Schematic representation of COVID-19 electrocautery plume experiment

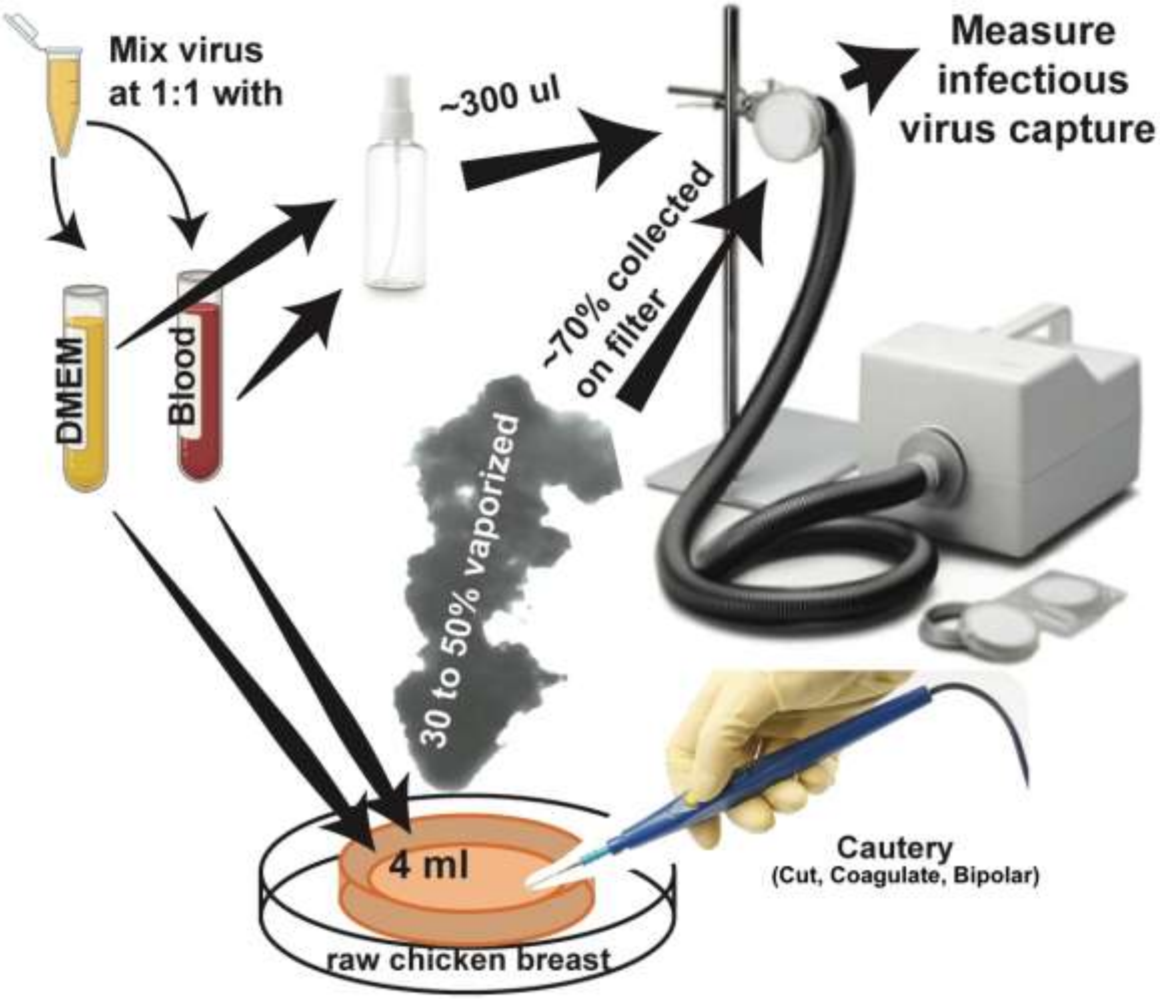

© 2021 Sowerby LJ et al. *JAMA Surgery.*
